# Construction sites as an important driver of dengue transmission: implications for disease control

**DOI:** 10.1186/s12879-018-3311-6

**Published:** 2018-08-08

**Authors:** Shaohong Liang, Hapuarachchige Chanditha Hapuarachchi, Jayanthi Rajarethinam, Carmen Koo, Choon-Siang Tang, Chee-Seng Chong, Lee-Ching Ng, Grace Yap

**Affiliations:** 1 Environmental Health Institute, 11, Biopolis Way, #06-05-08, Singapore, 138667 Singapore; 2 Environmental Public Health Operations Department, National Environment Agency, 40, Scotts Road, #13-00, Singapore, 228231 Singapore; 3 School of Biological Sciences, Nanyang Technological University, 60 Nanyang Drive, Singapore, 637551 Singapore

**Keywords:** Dengue, Environmental driver, Construction sites, Genotyping, Surveillance, Control

## Abstract

**Background:**

In 2013 and 2014, Singapore experienced its worst dengue outbreak known-to-date. Mosquito breeding in construction sites stood out as a probable risk factor due to its association with major dengue clusters in both years. We, therefore, investigated the contribution of construction sites to dengue transmission in Singapore, highlighting three case studies of large construction site-associated dengue clusters recorded during 2013–16.

**Methods:**

The study included two components; a statistical analysis of cluster records from 2013 to 2016, and case studies of three biggest construction site-associated clusters. We explored the odds of construction site-associated clusters growing into major clusters and determined whether clusters seeded in construction sites demonstrated a higher tendency to expand into major clusters. DENV strains obtained from dengue patients residing in three major clusters were genotyped to determine whether the same strains expanded into the surroundings of construction sites.

**Results:**

Despite less than 5% of total recorded clusters being construction site-associated, the odds of such clusters expanding into major clusters were 17.4 (2013), 9.2 (2014), 3.3 (2015) and 4.3 (2016) times higher than non-construction site clusters. *Aedes* premise index and average larvae count per habitat were also higher in construction sites than residential premises during the study period. The majority of cases in clusters associated with construction sites were residents living in the surroundings. Virus genotype data from three case study sites revealed a transmission link between the construction sites and the surrounding residential areas.

**Conclusions:**

Significantly high case burden and the probability of cluster expansion due to virus spill-over into surrounding areas suggested that construction sites play an important role as a driver of sustained dengue transmission. Our results emphasise that the management of construction-site associated dengue clusters should not be limited to the implicated construction sites, but be extended to the surrounding premises to prevent further transmission.

**Electronic supplementary material:**

The online version of this article (10.1186/s12879-018-3311-6) contains supplementary material, which is available to authorized users.

## Background

Dengue is a complex clinical syndrome with various epidemiological confounders that make disease control a challenging task. Dengue virus (DENV) transmission relies on three obligatory components: host (humans), vector (primarily *Aedes aegypti*) and the virus (DENV serotypes 1, 2, 3 and 4). Approximately 3.6 billion people are at risk of DENV infection, with an estimated 50–200 million infections occurring annually [1]. The large-scale re-emergence of dengue fever over the past few decades has raised a serious international public health concern, especially in the tropics and subtropics [2].

Located in the Asia-Pacific region, Singapore is not spared from dengue fever outbreaks despite vector control efforts since 1960s [3, 4]. In 2013 and 2014, the country experienced its worst dengue outbreak known-to-date, with 22,077 (seven deaths) and 18,318 confirmed cases (five deaths), respectively [5]. Several epidemiological, entomological and virological parameters have been analysed to determine the possible factors responsible for outbreaks [5–7]. Among many contributors, environmental factors appear to be one of the important influential components that can impact both human and vector populations [8]. Some of these environmental factors can be modified or manipulated to mitigate dengue transmission.

One of such potential environmental factors identified in Singapore is the condition of construction sites. Being an urban city with high population density (> 7000 people per metre square), Singapore undergoes regular upgrading of its infrastructure. In 2013, more than 2500 construction sites of different scales were recorded. Construction projects primarily rely on foreign labour, including workers from dengue non-endemic or newly endemic countries, who may be immunologically naïve to dengue. Due to the nature of construction work, these workers are unlikely to be permanently stationed, and tend to work at different sites across the country. As construction sites are dynamic environments, different phases of construction may allow the sites to be conducive for *Aedes* breeding when potential habitats are constantly created or not removed. For example, water puddles on various surfaces, such as concrete floors in uncompleted buildings, are common and have shown to be attractive breeding habitats for *Ae. aegypti* [9, 10]. It is highly challenging to manoeuvre among construction materials to properly survey and eliminate all potential breeding habitats. During 2013–15, several major dengue clusters were linked to construction sites [11–13]. Anecdotal construction site outbreaks in Singapore have been reported.

In the present study, we aimed to investigate the contribution of construction sites as an important environmental factor of dengue transmission in Singapore, and further examined this phenomenon through three case studies of large construction site-associated dengue clusters recorded during 2013–16.

## Methods

### Exploring the impact of construction site-associated cases on the cluster size

In Singapore, dengue cases are clustered for targeted vector control operational purposes based on their proximity and onset dates. All cases are laboratory-confirmed. A cluster is formed when two cases are located within 150 m apart from each other and their onset dates are within 14 days. Subsequent cases that fulfil the same criteria are also tagged to the same cluster. Clusters are further stratified into major and minor - a major cluster is defined as having 10 or more reported cases. All cases that do not belong to any cluster are defined as sporadic. When cases occur in a construction site within the boundary of a defined cluster, it is considered as a construction site-associated cluster. In the present study, we analysed the epidemiological records of dengue cases from 2013 to 2016. Clusters were categorised into construction site-associated and non-construction site-associated clusters. We explored the odds of construction site-associated clusters growing into major clusters, the average number of cases per large construction site-associated cluster, and determined whether clusters initiated by construction site-associated cases demonstrated a higher tendency to expand into major clusters. A cluster initiated by construction site-associated cases was defined as having any of the first notified cases being a worker from the construction site. The Odds ratio (OR) with 95% confidence interval was calculated according to Altman [14].

### Case studies: Bedok reservoir road, Choa Chu Kang and Tampines clusters

Bedok Reservoir Road (BR) and Tampines are located in the eastern region of Singapore, while Choa Chu Kang (CCK) is located in the western region, approximately 21 km apart. BR is a residential area of 2.4 km^2^ with 23,600 residents. CCK is a residential area of 6.1 km^2^ with 174,320 residents. There were 261,240 residents in Tampines within an area of 21 km^2^. All three clusters were classified operationally as construction site-associated clusters. BR cluster was active from EW35 to 47 in 2013 and recorded 158 cases. CCK cluster recorded 534 cases from EW24 to EW33 in 2014. Tampines cluster recorded 280 cases from EW42 of 2015 to EW6 of 2016. Analysing the epidemiological aspects of clusters, we attempted to find out their similarities and how they may have contributed to the establishment of major clusters. Additionally, DENV strains obtained from dengue patients among construction workers and residents living in BR, CCK and Tampines clusters were genotyped. Genotyping was carried out based on the phylogenetic analysis of envelope (*E*) gene sequences of DENV as described elsewhere [15].

## Results

### Overall case burden of construction site-associated clusters was significantly high

A summary of the cluster categories, distribution and case counts is given in Table 1. A cluster was defined as a congregation of two or more cases located within 150 m apart from each other in 14 days of onset from the last reported case. When cases occurred in a construction site within the boundary of a defined cluster, the cluster was operationally classified as a construction site-associated cluster. Of the 6568 clusters reported from 2013 to 2016, the number of major clusters (each consisting of 10 or more notified cases) ranged between 102 (5.9%, 2016) and 199 (11.7%, 2013). The highest number of major clusters in 2013 was due to the epidemic transmission [5]. Nevertheless, major clusters contributed to approximately half of the “clustered” cases in each year, except for 2016 (35.9%). This highlights the impact of major clusters on the overall dengue case burden in Singapore.

**Table 1:** Case details of major (≥10 cases) and minor (< 10 cases) clusters: Overall and construction site-associated clusters

	2013				2014				2015				2016			
Total no. of clusters	1705				1823				1319				1721			
No. of clusters based on size^a^	≥10		< 10		≥10		< 10		≥10		< 10		≥10		< 10	
	199 (11.7%)		1506 (88.3%)		133 (7.3%)		1690 (92.7%)		108 (8.2%)		1211 (91.8%)		102 (5.9%)		1619 (94.1%)	
Average cases per cluster	30.7		3.3		39.7		2.9		31.3		2.9		26.4		3.0	
Construction site-associated clusters	Yes		No		Yes		No		Yes		No		Yes		No	
	50 (2.9%)		1655 (97.1%)		88 (4.8%)		1735 (95.2%)		41 (3.1%)		1278 (96.9%)		50 (2.9%)		1671 (97.1%)	
Cluster size^a^	≥10	< 10	≥10	< 10	≥10	< 10	≥10	< 10	≥10	< 10	≥10	< 10	≥10	< 10	≥10	< 10
No. of clusters	33	17	166	1489	32	56	101	1634	9	32	99	1179	10	40	92	1579
No. of cases	1336	62	4777	4893	2473	216	2805	4707	537	102	2845	3391	360	135	2331	4661
Average cases per cluster	40.5	3.6	28.7	3.3	77.1	3.9	27.8	2.9	59.7	3.2	28.7	2.9	36	3.4	25.3	3.0
Odds ratio^b^ (95% CI)	17.4 (9.49–31.9)				9.24 (5.73–14.9)				3.35 (1.55–7.21)				4.29 (2.07–8.85)			

^a^≥10 = major clusters; < 10 = minor clusters

^b^Odds ratio of construction site-associated clusters expanding into major clusters. *CI* confidence interval

The construction-site associated major clusters recorded 1.41 (2013) - 2.77 (2014) times more cases per cluster than those that were not associated with construction sites. The overall case burden of construction site-associated clusters was significantly higher than that of remaining clusters (*p*-value < 0.001 for all 4 years).

### The probability of construction site-associated clusters expanding into major clusters was significantly high regardless of the origin of the initial case

Of all clusters reported from 2013 to 2016, the proportion of construction site-associated clusters was low and ranged from 2.9% (2013 and 2016) to 4.8% (2014) (Table 1). However, a substantially high proportion of construction site-associated clusters was major clusters (range: 20% in 2016–66% in 2013). The OR of construction site-associated clusters expanding into major clusters was significantly higher than the remaining clusters in each year (*p*-value < 0.01) (Table 1).

It was then, determined whether there was any relationship between the size of construction site-associated clusters and the origin of the first notified case (either a worker within the construction sites or a resident living in the vicinity of the construction sites), based on disease onset dates. The findings showed that the probability of expansion of a particular cluster into a major cluster was not dependent on the first notified case being a worker (Table 2). The OR of construction site-initiated clusters (the first case was a worker) expanding into major clusters was not significantly different from those that had residents as the first notified case (Table 2). The proportion of major clusters and the average number of cases per cluster was comparable between the construction site-initiated clusters and those initiated by residents in the vicinity (Table 2).

**Table 2:** Summary of construction site-associated clusters in which the first known case was notified from the construction site or from the surrounding areas

	2013				2014				2015				2016			
Total no. of clusters	50				88				41				50			
No. of clusters that notified the first case from a construction site^a^	Yes		No		Yes		No		Yes		No		Yes		No	
	35		12		54		28		25		12		31		9	
Cluster size^b^	≥10	< 10	≥10	< 10	≥10	< 10	≥10	< 10	≥10	< 10	≥10	< 10	≥10	< 10	≥10	< 10
No. of clusters	24	11	9	3	19	35	11	17	6	19	3	9	6	25	3	6
No. of cases	1038	44	298	10	1546	112	836	54	425	59	112	30	231	79	107	25
Average cases per cluster	43.3	4	33.1	3.3	81.4	3.2	76.4	3.2	70.8	3.1	37.3	3.3	38.5	3.2	35.7	4.2
Odds ratio^c^ (95% CI)	0.73 (0.16–3.22)				0.84 (0.33–2.15)				0.95 (0.20–4.68)				2.08 (0.4–10.8)			

^a^Numbers given here are less than the total number of clusters stated because of the non-availability of index case information from certain clusters

^b^≥10 = major clusters; < 10 = minor clusters

^c^Odds ratio of construction site-associated clusters expanding into major clusters. CI = confidence interval

We, next, investigated whether the scale of a particular construction site has any impact on the final size of construction site-initiated clusters. We used the estimated cost of respective construction projects as a proxy for their scale and classified sites costing 10 million Singapore dollars and above as large-scale projects. The OR of clusters initiated within large-scale construction sites expanding into major clusters was not statistically significant in each year (Table 3).

**Table 3:** Summary of construction site-initiated^a^ clusters based on the scale of construction projects^b^

	2013				2014				2015				2016			
Scale of construction site	Large		Small		Large		Small		Large		Small		Large		Small	
	12		18		15		40		13		18		17		11	
Cluster size^a^	≥10	< 10	≥10	< 10	≥10	< 10	≥10	< 10	≥10	< 10	≥10	< 10	≥10	< 10	≥10	< 10
No. of clusters	9	3	12	6	6	9	16	24	2	11	4	14	4	13	4	7
Odds ratio^b^ (95% CI)	1.5 (0.29–7.68)				1.0 (0.30–3.36)				0.64 (0.10–4.14)				0.54 (0.10–2.84)			

^a^Construction site-initiated clusters are those that notified the first case among construction site workers

^b^Constructions sites costing ten million Singapore dollars and above were classified as large scale projects

### Virus exchange between the construction sites and surrounding residential areas is likely to drive the rapid expansion of clusters

**Fig. 1:**
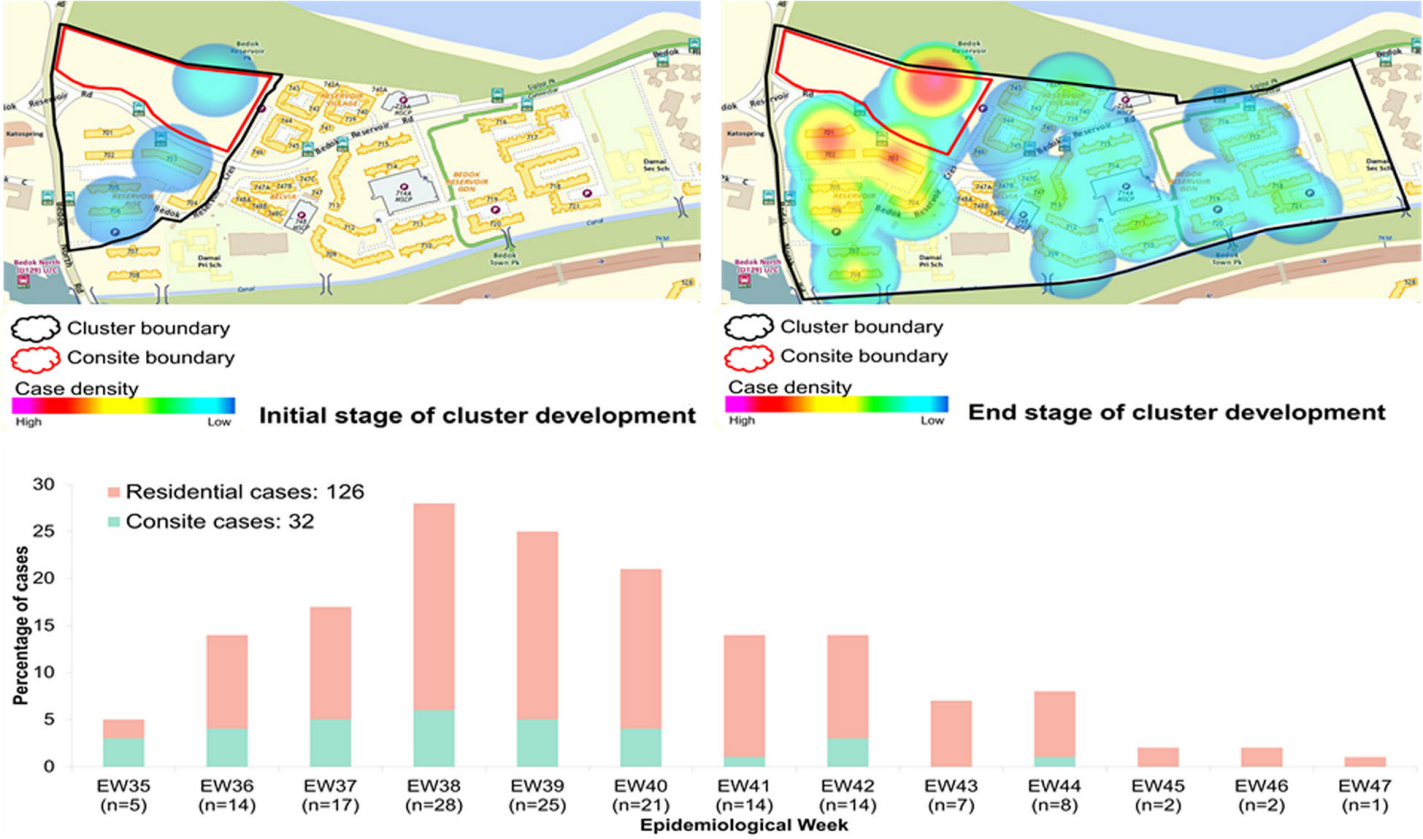
Spatial case density map and temporal distribution of reported dengue cases in Bedok Reservoir Road cluster. The size of the cluster is 0.39 km^2^. Cluster boundary is shown in black and the construction site boundary in red. The spatial fluctuations of case density during the initial and end stages of the cluster are shown in different shades of colour as per the legend. The graph below the maps shows the weekly distribution of cases among residents and construction site workers. Consite = construction site

**Fig. 2:**
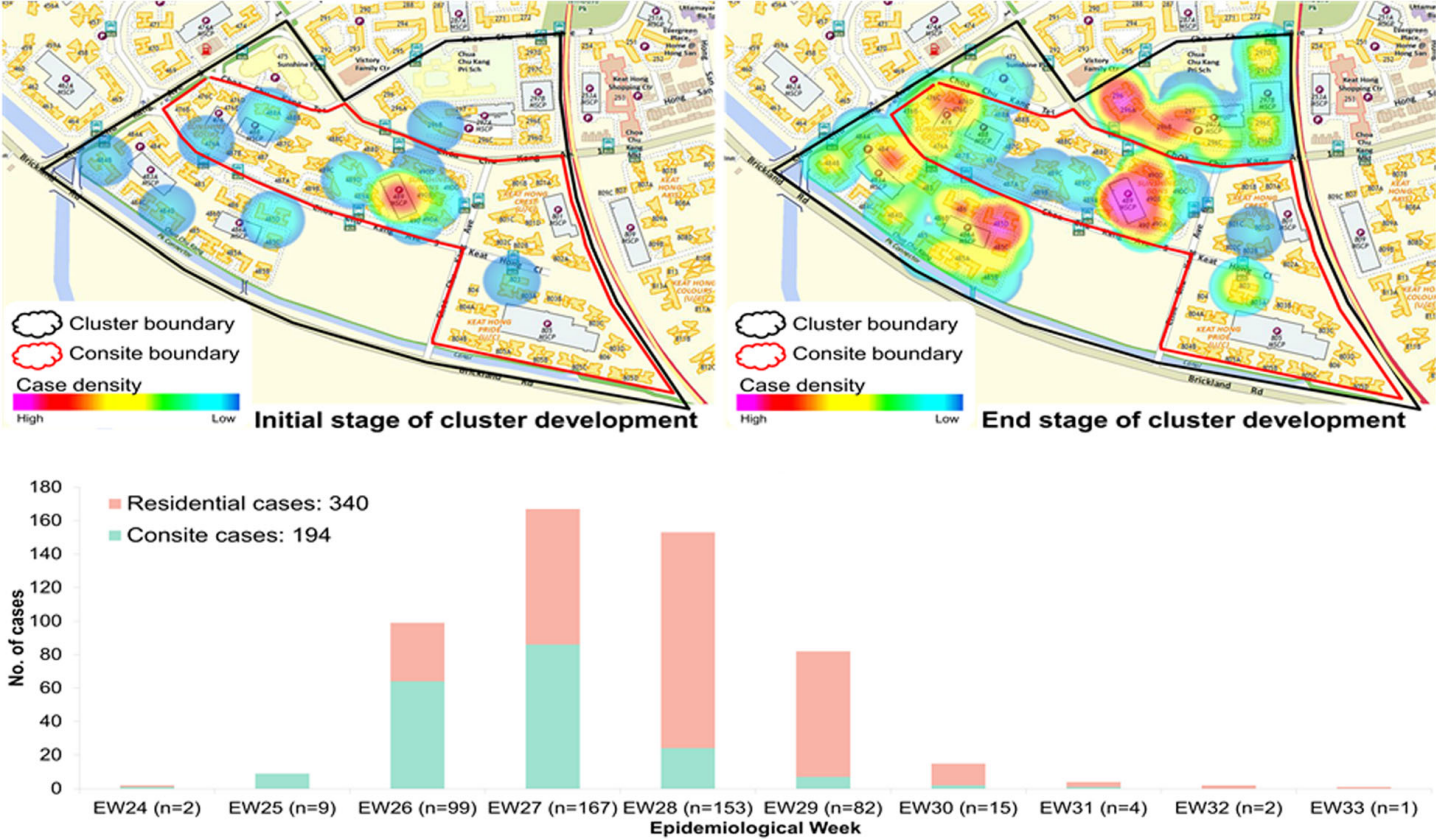
Spatial case density map and temporal distribution of reported dengue cases Choa Chu Kang cluster. The size of the cluster is 0.46 km^2^. Cluster boundary is shown in black and the construction site boundary in red. The spatial fluctuations of case density during the initial and end stages of the cluster are shown in different shades of colour as per the legend. The graph below the maps shows the weekly distribution of cases among residents and construction site workers. Consite = construction site

**Fig. 3:**
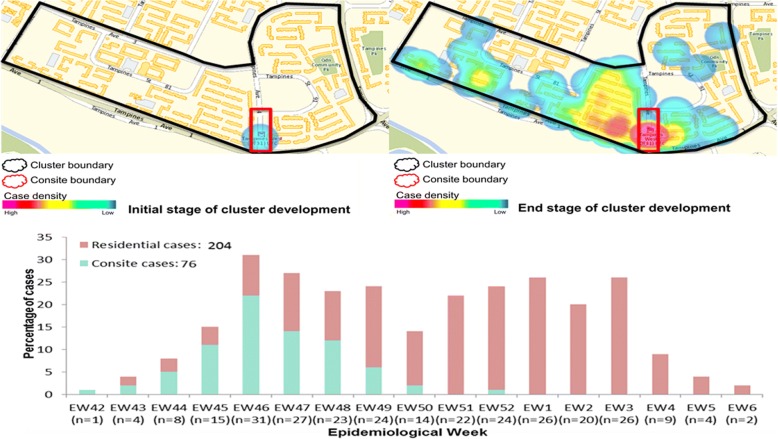
Spatial case density map and temporal distribution of reported dengue cases Tampines cluster. The size of the cluster is 0.7 km2. Cluster boundary is shown in black and the construction site boundary in red. The spatial fluctuations of case density during the initial and end stages of the cluster are shown in different shades of colour as per the legend. The graph below the maps shows the weekly distribution of cases among residents and construction site workers. Consite = construction site

We further examined the contribution of construction sites as an important driver of dengue transmission through three case studies of construction site-associated major dengue clusters; Bedok Reservoir Road (BR) in 2013 (Fig. 1), Choa Chu Kang (CCK) in 2014 (Fig. 2) and Tampines during 2015–16 (Fig. 3). These study sites were chosen based on the case burden. Each site recorded the highest number of cases among construction site associated clusters in respective years. The index cases of BR and Tampines clusters were recorded in respective construction sites, whereas that of the CCK cluster was a resident in the neighbouring area. Of 158 cases recorded in the BR cluster, 20.3% of cases were workers from a construction site located within the cluster boundary. Similarly, 36.3% and 27.1% of cases in CCK (*n* = 534) and Tampines (*n* = 280) clusters respectively were among site workers. The spatio-temporal case density analysis showed that the transmission was persistent within each construction site and gradually intensified during the active period of respective clusters (Figs. 1, 2 and 3). We compared the envelope gene (*E*) identity of virus strains (*n* = 111) obtained from construction workers and residents in each study site (BR; *n* = 35, CCK; *n* = 68 and Tampines; *n* = 8) to determine whether the expansion of respective clusters was due to spilling over of similar virus strains into surrounding areas.

**Fig. 4:**
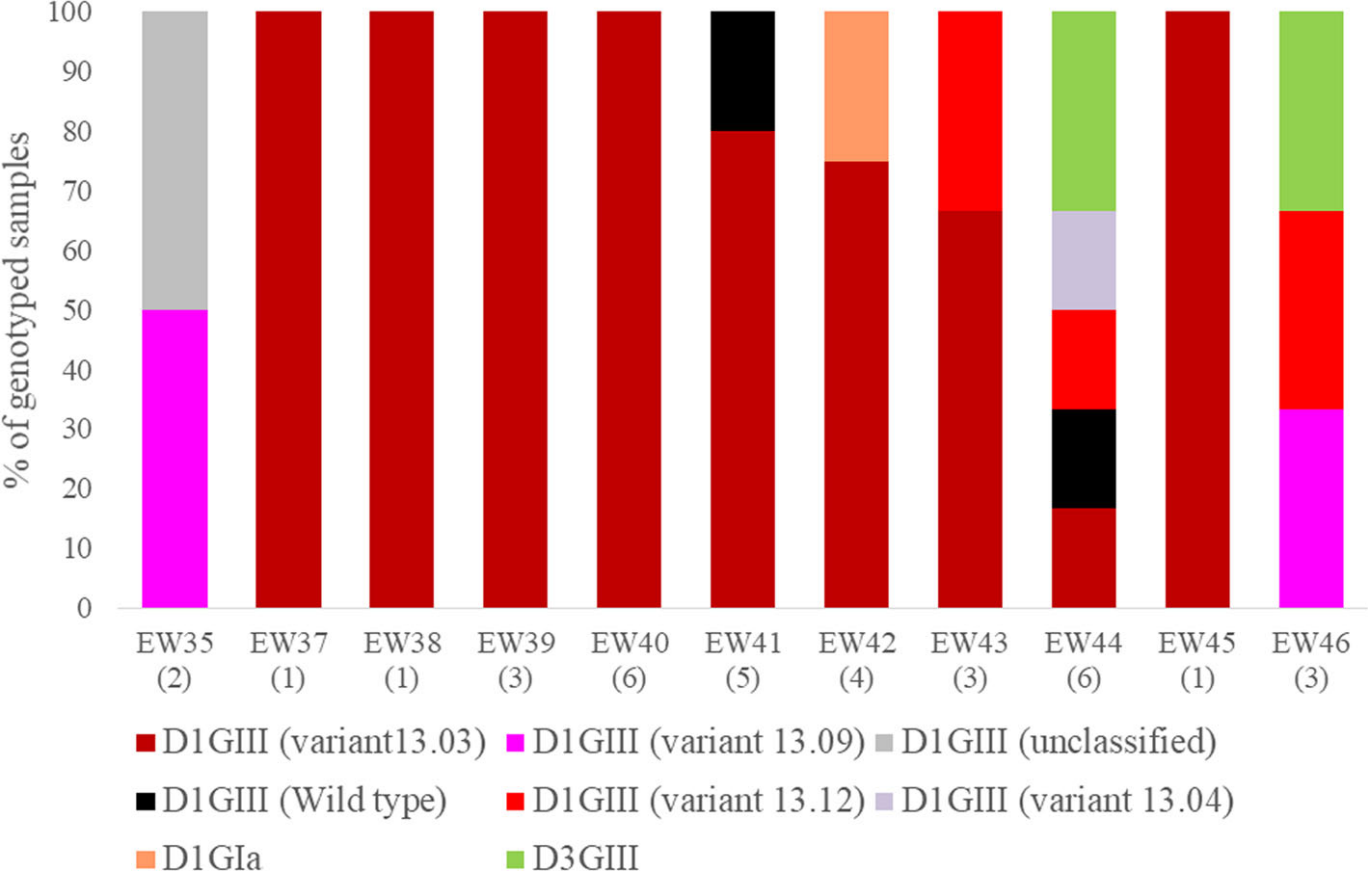
Temporal pattern of DENV diversity in Bedok Reservoir Road cluster. The data is shown for the weeks during which virus strains were genotyped. The actual number of samples genotyped per week is given in brackets on the x-axis. The variants of DENV-1 [7] and different types of virus strains [5] have been described elsewhere. D1 = DENV-1; D3 = DENV-3; GI = genotype I; GIII = genotype III; unclassified = variant classification is not available

Of 35 viruses analysed in BR cluster, DENV-1 (*n* = 32) was the most common serotype. The remaining three were DENV-3 viruses. Amongst the DENV-1 strains, 31 viruses (96.9%) belonged to DENV-1 genotype III, which was the dominant lineage during the 2013–2014 epidemic [5]. The remaining sequence belonged to DENV-1 genotype I. DENV-1 genotype III strains were detected throughout the entire transmission period of BR cluster (Epidemiological week (EW) 35–47), indicating their consistent presence in the cluster (Fig. 4). Moreover, those 31 DENV-1 genotype III strains shared high nucleotide (99.4–100%) and amino acid (99.3–100%) similarities. Of them, 22 (71%) sequences belonged to the same variant (13.03) [7]. Interestingly, virus diversity increased during the late phase of the active period of BR cluster (EW44–46), during which DENV-3 was also detected, indicating multiple introduction events towards the end of transmission period (Fig. 4).

**Fig. 5:**
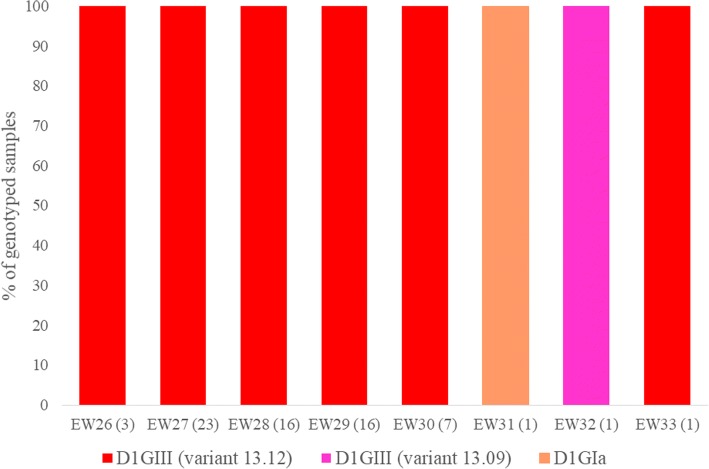
Temporal pattern of DENV diversity in Choa Chu Kang cluster. The data is shown for the weeks during which virus strains were genotyped. The actual number of samples genotyped per week is given in brackets on the x-axis. The variants of DENV-1 [7] and different types of virus strains [5] have been described elsewhere. D1 = DENV-1; GI = genotype I; GIII = genotype III

All virus strains (*n* = 68) genotyped in CCK cluster belonged to DENV-1. Among them, 67 (98.5%) strains were of genotype III and the remaining strain belonged to genotype I. Likewise in BR cluster, DENV-1 genotype III strains in CCK cluster were closely related (nucleotide similarity of 99.5–100% and amino acid similarity of 99.1–100%) throughout the entire period of the cluster (Fig. 5). Sixty-six (98.5%) sequences of DENV-1 genotype III belonged to the same variant (13.12) [7].

**Fig. 6:**
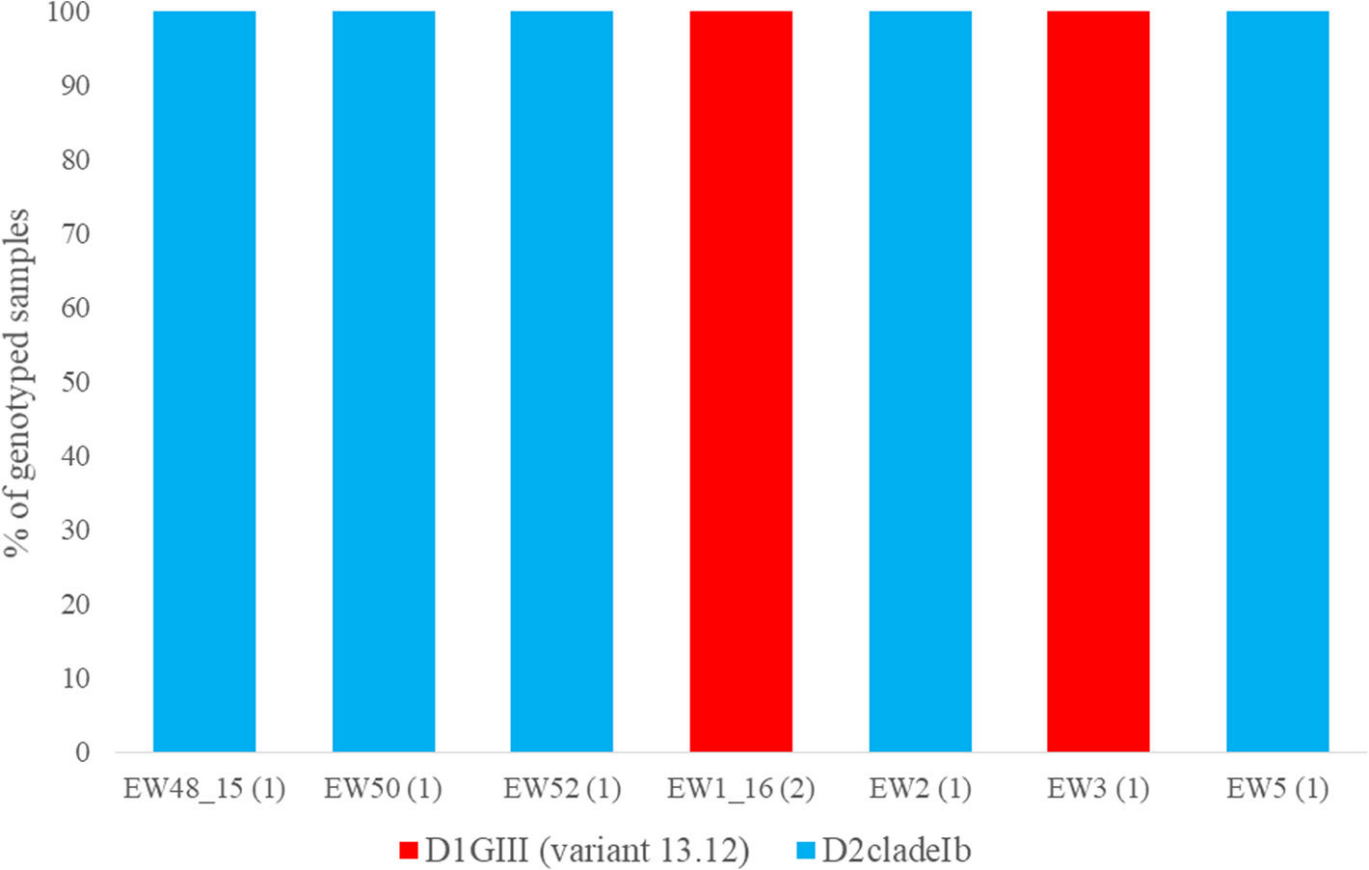
Temporal pattern of DENV diversity in Tampines cluster. The data is shown for the weeks during which virus strains were genotyped. The actual number of samples genotyped per week is given in brackets on the x-axis. The variants of DENV-1 [7] and different types of virus strains [5] have been described elsewhere. D1 = DENV-1; D2 = DENV-2; CladeIb = cosmopolitan clade Ib; GIII = genotype III

On the other hand, a mixed serotype pattern was observed in the Tampines cluster (Fig. 6). Of eight viruses genotyped in the cluster, five (62.5%) belonged to DENV-2 cosmopolitan genotype (clade Ib; [5, 7]) and the remaining viruses belonged to DENV-1 genotype III. Nevertheless, all DENV-2 and DENV-1 virus sequences were closely related and clustered within the same variants of respective serotypes. Four out of five DENV-2 and two out of three DENV-1 viruses shared identical *E* gene sequences.

In summary, the virus populations in all three study sites were highly homogenous. The dominant strains were introduced at the beginning of the transmission period and circulated throughout the active period of each cluster. These observations indicated that the transmission has primarily been driven by a single introduction event at each study site and the expansion of clusters resulted from spill-over transmission in surrounding areas. Our previous virus genotype data has suggested the exchange of identical virus strains between distant construction sites managed by the same construction company (unpublished data, Environmental Health Institute). Therefore, repeated introduction of the same variants from other distant sites cannot be completely ruled out.

## Discussion

The overall case burden of construction site-associated clusters was significantly higher than that of remaining clusters and the probability of construction site-associated clusters expanding into major clusters was significantly high. These findings demonstrated the potential impact of construction sites on dengue transmission, especially when spill-over transmission takes place in surrounding residential areas, where the population may have relatively low herd immunity [6, 16]. However, the ultimate size of clusters was not dependent on the size of the construction project or whether the cases initially occurred within the construction sites. Our case studies highlighted the likelihood of virus exchange between the construction sites and the immediate periphery that subsequently resulted in further expansion of clusters. The high homogeneity of dominant virus populations among construction workers and residents over the active period of study site clusters indicated potentially a strong transmission link between construction sites and residential areas. The fact that a higher proportion of cases in our case studies was detected among residents than the construction workers suggested the possibility of a two-way virus exchange between construction sites and surrounding areas once the transmission established.

These observations implied that the expansion of clusters was not purely attributable to cases in construction sites, but also due to the external neighbouring environment being conducive for virus transmission. Because the workers move around in the neighbourhood and mosquitoes could migrate between the construction sites and the external periphery, it is difficult to cease new cases until the transmission is interrupted in both environments. Nevertheless, as the workers are often highly congregated in construction sites, the disease tends to spread faster among workers than the residents. It has previously been reported that due to poor house-keeping, construction sites tend to create an environment conducive for mosquito breeding [17]. In the presence of suitable tropical climatic factors [18], coupled with the dynamic nature of construction sites, breeding habitats can be easily established and often be overlooked .

National Environment Agency (NEA)‘s inspection statistics show that construction sites have the propensity of breeding mosquitoes with high larval density (Additional file 1: Figure S1 and Additional file 2: Table S1). As such, construction sites, where mosquito breeding is detected or have poor housekeeping face stringent penalties, including Stop Work Orders and prosecution in Court. In addition, NEA has introduced additional measures since 2015 to enhance dengue control efforts in construction sites:

- formation of dedicated teams for the inspection of construction sites for mosquito breeding. The inspection frequency at large construction sites has been stepped up from quarterly to monthly.
- publishing the list of Stop Work Order sites to serve as a deterrent to contractors to maintain good housekeeping. The publication will also encourage contractors to promptly take actions to remove conditions favourable for mosquito breeding and to limit the potential of secondary infections in the area.
- temperature screening of workers at construction sites in areas of dengue transmission to enable early case detection, application of insect repellents on both dengue-infected and healthy workers and quarantining infected workers under bed nets or in air-conditioned sick bays to prevent further transmission.

NEA has also implemented the Environmental Control Officers Scheme to mandate companies to take up the responsibility of minimizing vector breeding at large construction sites and protecting the staff from acquiring vector-borne diseases. The role of Environmental Control Officers is critical in preventing the initiation of new clusters.

One of the limitations of our study is the non-availability of entomological data to characterise mosquito density fluctuations at construction sites during different construction phases to highlight specific risk factor(s) for more targeted vector control operations. Without entomological data, it is difficult to pinpoint whether the relatively high transmission intensity of DENV in construction-site associated clusters is due to the abundance of vectors. Nevertheless, our data on higher premise index and more breeding habitats may be used as a proxy for higher vector abundance in construction sites than residential premises. On the other hand, high transmission could be driven by a continual source of viruses introduced into these sites through the labour-force as shown in our findings. The successful establishment of transmission is also determined by the immunological status of construction workers. Therefore, the source of transmission in construction sites seems to be multi-factorial and opportunistic. Another limitation is the lack of information on the actual size of individual construction sites. We investigated whether the size of the construction sites impacts on the final size of clusters by using the estimated cost of the projects as a proxy. However, the data was not available for all construction sites during the study period. Moreover, the estimated project cost not only depends on the land area, but also on the land value that varies based on the location.

## Conclusions

The role of construction sites as an important driver of dengue transmission cannot be underestimated. Despite an intensive island-wide vector control program in Singapore, vector surveillance in construction sites is still a challenge given the large number of construction activities at any one time. The findings emphasise that dengue control measures of construction site-associated clusters should not be limited to the sites per se, but to stretch out to a “buffer-zone” in the surrounding residential areas to minimize sustained virus transmission. Further studies are warranted to characterise the mosquito breeding and population density fluctuations at sites during different phases of the construction work. As many dengue endemic countries in Asia and South/Central America have emerging economies and are undergoing rapid infrastructure upgrading, our findings have important implications for the policy planning and control of dengue in an era of rapid urbanisation.

## Additional files

Additional file 1: **Figure S1.** *Aedes* Premises Index of government housing (HDB) flats, private apartments, landed properties, construction sites and dormitories (2013–2016). Premises Index is defined as the number of inspected premises found with *Aedes* breeding out of 100 inspected premises. (TIF 14 kb)

Additional file 2: **Table S1.** Mosquito breeding habitats and larvae counts detected in construction sites and residential premises from 2013 to 2016 (DOCX 12 kb)

## Abbreviations

BR: Bedok Reservoir Road
CCK: Choa Chu Kang
DENV: Dengue virus
E: Envelope gene
EW: Epidemiological week
NEA: National Envioronment Agency
OR: Odds ratio
