# Supplementary figures and images for: Construction sites as an important driver of dengue transmission: implications for disease control
Source: BMC Infect Dis. 2018 Aug 8;18:382. doi: 10.1186/s12879-018-3311-6 (PMC6083507; doi:10.1186/s12879-018-3311-6)

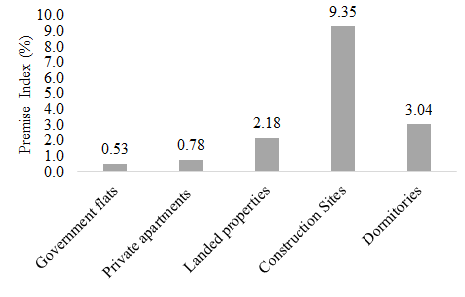

Supplement: Supplementary file 1 — Figure S1. Aedes Premises Index of government housing (HDB) flats, private apartments, landed properties, construction sites and dormitories (2013–2016). Premises Index is defined as the number of inspected premises found with Aedes breeding out of 100 inspected premises. (TIF 14 kb) [file 12879_2018_3311_MOESM1_ESM.tif]
