# Supplementary material for: Construction sites as an important driver of dengue transmission: implications for disease control
Source: BMC Infect Dis. 2018 Aug 8;18:382. doi: 10.1186/s12879-018-3311-6 (PMC6083507; doi:10.1186/s12879-018-3311-6)
Supplement: Supplementary file 2 — Table S1. Mosquito breeding habitats and larvae counts detected in construction sites and residential premises from 2013 to 2016 (DOCX 12 kb) [file 12879_2018_3311_MOESM2_ESM.docx]

Additional Table 1. Mosquito breeding habitats and larvae counts detected in construction sites and residential premises from 2013 to 2016

| Premises Type | Breeding Habitats | Larvae Count | Average Larvae Count per Breeding Habitat | Ratio of average larvae count per breeding habitat (with residential premises as the Base) |
| --- | --- | --- | --- | --- |
| Construction Sites | 4,989 | 222,227 | 44.5 | 1.84 |
| Residential Premises | 3,854 | 917,616 | 24.2 | 1 |
